# An observational study on the sub-acute effects of mephedrone on mood, cognition, sleep and physical problems in regular mephedrone users

**DOI:** 10.1007/s00213-018-4953-1

**Published:** 2018-06-26

**Authors:** Lina Homman, Jessica Seglert, Michael J. Morgan

**Affiliations:** 1 Department of Psychology, University of Sussex, Brighton, BN1 9RH UK; 2 Center for Social and Affective Neuroscience, Linköping University, 581 83 Linköping, Sweden; 3 Norwegian Center for Addiction Research, University of Oslo, 0315 Oslo, Norway

**Keywords:** Mephedrone, Cathinone, Sub-acute effects, Mood, Cognition, Sleep, Physical problems

## Abstract

**Rationale:**

Mephedrone (4-methylmethcathinone; 4-MMC) is a novel recreational drug similar to methylenedioxymethamphetamine (MDMA) and amphetamine. Several adverse effects have been reported, but little is known about its sub-acute effects.

**Objectives:**

To study sub-acute effects of mephedrone over a period of 9 days.

**Methods:**

Recreational mephedrone users were recruited and followed over a time period of 9 days. It was recorded whether participants consumed mephedrone or not within the period of testing; those who did were compared to those who did not. Forty-six regular mephedrone users (22 males, 24 females) participated, 21 participants voluntarily opted to consume mephedrone 1–3 days after baseline and 25 opted to abstain. Participants were assessed at baseline on a multitude of measures and provided daily reports on cognition, sleep, mood, physical problems, mephedrone cravings and substance use on each subsequent day of the study. The study controlled for psychopathology, sleep, past and current substance use, impulsivity and demographics.

**Results:**

Those who consumed mephedrone reported persistent negative mood, physical problems and fatigue, compared to those who did not—after controlling for baseline group differences in sleep and subsequent alcohol and cannabis use.

**Conclusions:**

The results provide the first prospective evidence of the duration and extent of specific undesirable sub-acute effects of mephedrone in regular recreational users and indicate sub-acute effects of mephedrone on mood, fatigue and physical symptoms.

## Introduction

### Mephedrone

Mephedrone (4-methylmethcathinone; “meow-meow”, “MCAT”, “bubbles”, “drone”) is a novel psychoactive substance (NPS), a cathinone analogue of methylamphetamine, structurally related to the naturally occurring phenylpropylamine alkaloid cathinone found in the khat plant (United Nations 2009; Advisory Council of Misuse of Drugs (ACMD) 2010; Dargan et al. 2010; Griffith et al. 2010; Morris 2010). NPS are a constantly changing class of substances and have modified the drug scene (EMCDDA 2014). Mephedrone appeared on the drug scene prior to the coining of the term NPS and have been around since the mid-2000 under the name of bath salts/plant food (McElrath and O’Neill 2011). The availability of mephedrone for legal purchase online, combined with a reduction in the availability and quality of other drugs (e.g. 3,4-methylenedioxymethamphetamine (MDMA) and cocaine), and widespread media coverage contributed to a marked increase in mephedrone consumption in Europe and the UK between 2009 and 2010 (Davey et al. 2010; Measham et al. 2010; Brunt et al. 2011). Prevalence of use has since decreased with a reduction from 4.4% in 2010/2011 to 0.5% in 2017 among 16 to 24-year-olds (EMCDDA 2017). The reduction in use is possibly partly due to the legal status of mephedrone which was controlled as a class B substance in the UK in April 2010 and throughout the European Union in December 2010 (European Monitoring Centre for Drugs and Drug Addiction (EMCDDA) 2010). Additional to this, the Psychoactive Substance Act of 2016 (British Home Office 2016) restricting the production, sale and supply of new psychoactive substances.

### Acute effects of mephedrone

Mephedrone is frequently compared to MDMA and amphetamines based on their similar acute effects (Kehr et al. 2011; Jones et al. 2016; Papaseit et al. 2016). A naturalistic study in humans indicated that mephedrone acutely impaired working memory, enhanced psychomotor speed and primed a marked increase in “wanting” for the drug (Freeman et al. 2012). A blind controlled study also found enhanced psychomotor speed and impairment of spatial short-term memory following acute mephedrone use (De Sousa Fernandes Perna et al. 2016). Other surveys, forums and reviews indicate acute symptoms of mephedrone to be elevated mood, euphoria, improved concentration, increase processing speed, talkativeness, increased energy, jaw-clenching, increased empathy, decreased appetite, increased confidence and appreciation for music (Dargan et al. 2010; Winstock 2010; Winstock et al. 2011a; Karila et al. 2016). The more common adverse effects involve cardiac, neurological and psychiatric effects such as agitation (Prosser and Nelson 2012). However, it is commonly noted in all these studies that effects of other drugs and polydrug use may account for the observed or reported symptoms. While the acute positive effects of mephedrone often are rated to be very similar to those of MDMA (Kapitány-Fövény et al. 2013), the acute negative effects are generally rated more negatively than MDMA (Matthews et al. 2017).

The similarities of mephedrone to MDMA and amphetamine have recently been extended from descriptive acute effects to also include clinical and psychopharmacological aspects. The first study to evaluate the clinical pharmacology of mephedrone in comparison to MDMA (Papaseit et al. 2016) found similarities to MDMA in regard to euphoria, well-being and changes in perception. Findings also indicated that the effects peaked earlier and lasted for a shorter duration than for MDMA, possibly contributing to the compulsive consumption patterns reported by users. Another study compared the acute effects of administration of mephedrone to MDMA and amphetamine in rats (Kehr et al. 2011). Results indicated that neurochemical and functional properties were similar to those of MDMA but also showed a rapid release and elimination of dopamine—properties similar to amphetamine.

### Sub-acute effects of mephedrone

Currently, relatively little evidence has been published on the sub-acute effects of mephedrone. Published work on the sub-acute effects of mephedrone is mainly derived either from surveys of recreational users (Dargan et al. 2010; Winstock 2010; Carhart-Harris et al. 2011; Winstock et al. 2011b) or clinical reports of mephedrone toxicity (Wood et al. 2010b, 2010a; Dargan et al. 2011; Schifano et al. 2011). A naturalistic study in humans reported impaired performance on tests of verbal memory and verbal fluency 48 h after drug consumption (Herzig et al. 2013). However, the study did not provide the basis for a daily assessment of the persistence of the sub-acute effects of mephedrone. A recent paper investigated acute and sub-acute effects (across the 7 days following use) of mephedrone and MDMA in young recreational users (Jones et al. 2016). Results showed more severe adverse effects of mephedrone compared to MDMA with increased levels of negative mood, issues with anxiety, anger and sleeping as well as increased cravings for mephedrone and paranoia in the days following use. However, the study was cross-sectional and the data retrospective. Furthermore, the study did not control for other possible confounding variables such as psychopathology, other substance use and lack of sleep due to use. In general, the results on sub-acute or similar to sub-acute effects of mephedrone indicate effects comparable to those of amphetamine and ecstasy (Prosser and Nelson 2012; Karila et al. 2015, 2016) or more adverse than those of ecstasy (Jones et al. 2016). Commonly reported sub-acute effects of MDMA and amphetamine are cognitive impairment, negative mood (depression and anxiety), paranoia, impaired ability to concentrate, physical problems and fatigue (Williamson et al. 1997; Verheyden et al. 2003; Huxster et al. 2006).

### Present study

In the present study, we aimed to investigate the sub-acute effects of mephedrone by recruiting recreational mephedrone users and monitor them over a set period in order to investigate whether those who did take mephedrone during this time differed from those who did not. The sample was divided into two groups based on whether they voluntarily consumed mephedrone 1–3 days after baseline. A thorough assessment was performed at baseline to see whether the two groups differed on for example dependence, craving, impulsivity and psychopathology. Participants subsequently completed daily assessments of mood, cognition, sleep, physical and psychological problems and craving for mephedrone over the total of 9 days (day of baseline and the succeeding 8 days). All other substance use during the post-baseline assessment period was monitored to control for possible effects of polydrug use. This design allowed us to differentiate the genuine sub-acute effects of mephedrone from its chronic effects and the sub-acute effects of any other substances.

## Method

### Procedure

The current study investigated sub-acute effects of mephedrone among regular mephedrone users daily over a period of 9 days. A mixed within and between subject design was employed to compare two groups of mephedrone users—those who opted to take mephedrone after baseline assessment (*N* = 21) versus those that opted to remain abstinent throughout the time period of the study (*N* = 25). Participants were recruited in 2010 through email advertisement at the University of Sussex, by flyer distribution in nightclubs and at the University and by using the “snowball” procedure (Solowij et al. 1992). A cash incentive was provided where one participant could win £50. The study was approved by the University of Sussex Psychology Department Ethics Committee.

Participants were eligible if they were regular recreational mephedrone users who reported using at least once a month and within the month prior to testing. All participants reported to not be under the influence of any substance at the time of interview. Participants were excluded if they reported current mental health disorders, current or past treatment for a mental health or substance abuse problem (*N* = 2). If participants were eligible, an interview with the researcher was arranged where baseline measures were assessed. Informed consent was obtained at the time of interview.

Participants were interviewed on a Thursday where baseline measures were assessed. Each participant was also given nine envelopes containing a Daily Rating Scale (DRS). The experimenter contacted each participant via text messages or phone call on the same evening and every following evening during the coming 8 days (total of 9 days) to ensure daily completion of the DRS. Participants confirmed that they had filled out the form via text message or phone call. Participants were requested to seal the completed DRS forms in coded envelopes at the end of each day to preclude the possibility of cross-checking their responses. At the end of the assessment period, the DRS measures were collected, and participants were debriefed. Out of the 21 participants who opted to use mephedrone during the testing period, 4 opted to use it on the day of baseline assessment (Thursday), 7 used it the day after (Friday) and 10 on the Saturday. Thus, the data of the DRS was coded so that baseline (Thursday) indicated baseline prior to mephedrone use and day 1 indicated the day after use (Friday/Saturday/Sunday), resulting in a total of seven measurement points (baseline and days 1–6). Eight additional participants attended initial baseline assessment but did not complete the study and were therefore excluded.

### Materials

At baseline, demographic information, general drug use history, a Mephedrone Questionnaire, a Mephedrone Craving Questionnaire, the SCL-90-R, BIS-11, FAST, and a Monetary-Choice Questionnaire were assessed. Alcohol use was assessed through the four-item Fast Alcohol Screening Test (FAST) (Hodgson et al. 2002). Past and current substance use was assessed with a questionnaire that requested participants to indicate if they had used a substance, and if so, the age of first use, time since last use, frequency of use and usual dose per session (Morgan 1998). Mephedrone craving was assessed with a 40-item questionnaire adapted from the Desires for Speed Questionnaire (James et al. 2004). A specific Mephedrone Questionnaire that included nine VAS items relating to attitudes to mephedrone, and seven items based on the DSM-IV-TR criteria for substance dependence was also administered. The latter symptoms served as a proxy measure for estimating the prevalence of mephedrone dependence (Looby and Earleywine 2007). Psychopathology was assessed with the SCL-90-R (Derogatis et al. 1973), a 90-item psychiatric symptom checklist. Trait impulsivity was assessed with the BIS-11, a 30-item impulsivity questionnaire (Patton et al. 1995). Delay discounting, a measure of how much value of a reward decreases with a delay to the reward, was assessed with the Monetary-Choice Questionnaire (Kirby et al. 1999), a 27-item questionnaire measure of delayed discounting. delay discounting is of interest when investigating substance use as delay discounting is associated with impulsiveness and both tend to be higher in individuals who regularly use a substance (Kirby et al. 1999). Missing data on baseline assessments was very low (97–99% response rate across measures) as the assessment was done together with the interviewer. Finally, daily assessment of mood, cognition, hunger, sleep, physical and psychological problems, mephedrone cravings and substance consumption (including any prescription drugs) after baseline was conducted with a version of the Daily Rating Scale (DRS), adapted from the original “six-item daily self-report measure assessing identified depressive domains” (Parker and Roy 2003). Participants were asked to record the time and date and report how they felt at their worst on each day on a 10-point Likert scale from “not at all” to “extremely”. Missing data was low (97, 96, 99, 95, 98, 99% response rate across the six items across days). Where missing data was present, pairwise deletion was applied.

### Statistical analyses

Group differences at baseline were assessed using chi-square tests and independent sample *t* tests. A factor analysis using varimax rotation was performed on the mephedrone craving questionnaire. Two-way repeated measures ANOVA, with drug group (control vs mephedrone) as the between factor and DRS assessment days as the within factor, was employed to determine whether mood, physical problems, cognition, sleep, craving for mephedrone, hunger and other substance use other than mephedrone, differed between groups over the testing period. Significant sphericity and multiple hypothesis testing were controlled for where appropriate. Potential confounding variables were treated as covariates. Analyses were performed using SPSS.

## Results

The study was completed by 46 individuals (22 males, 24 females) aged 19–36 (M = 23.76, SD = 3.45). Twenty-one participants voluntarily opted to take mephedrone 0–2 days after initial drug-free baseline testing (mephedrone group), while the remaining 25 participants opted to abstain from mephedrone for this entire period (control group).

### Baseline measures across the two groups

Descriptive statistics of demographics, BIS-11, SCL-90-R, delayed discounting, DSM dependence, FAST and smoking status are presented in Table 1. No significant group differences were observed on any measure.

**Table 1:** Personal details by group

Characteristics	Control (*N* = 25)	Mephedrone (*N* = 21)	Group difference
Female (*N*)	12	12	*χ*^2^ (1) = .38, *p* = .54
Age (M, SE)	23.15 (.65)	24.48 (.79)	*t*(44) = 1.30, *p* = .20
Education (*N*) (GCSE/A-levels/university)	1/11/13	0/4/17	*χ*^2^ (2) = 4.48, *p* = .11
Smoker	23	19	*χ*^2^ (1) = .03, *p* = .86
FAST (M, SE)	9.92 (.39)	9.81 (.60)	*t*(44) = .16, *p* = .87
BIS-11 attentional	2.28 (.05)	2.21 (.05)	*t*(44) = .93, *p* = .36
BIS-11 motor	2.49 (.18)	2.29 (.07)	*t*(44) = .94, *p* = .35
BIS-11 non-planning	2.66 (.08)	2.57 (.07)	*t*(44) = .82, *p* = .42
SCL-90-R	.75 (.11)	.73 (.09)	*t*(44) = .17, p = .87
Delayed discounting	12.96 (.43)	12.14 (.68)	*t*(44) = 1.04, *p* = .30
DSM-IV criteria for dependence	2.88 (.24)	3.24 (.28)	*t*(44) = .97, *p* = .34

*FAST* Fast Alcohol Screening Test, *BIS-11* trait impulsivity, *SCL-90-R* psychopathology.

### Substance use prior to study

No significant differences between groups were found regarding substance consumption in the last year (Table 2) or of participants’ mephedrone consumption history (Table 3). None of the participants reported taking mephedrone within the 4 days prior to baseline testing. However, one participant reported taking ecstasy 2 days prior to baseline. The participant was removed from all analyses to assess whether a significant effect was observed. It was not, and the participant remained in the analyses. All participants reported alcohol use within the previous 4 days. This was not correlated to whether participants chose to consume mephedrone nor any of the sub-acute effects. Twenty-four participants reported cannabis use within the past 4 days, 11 of who were in the control group and 13 in the mephedrone group. Significant correlations were observed between number of days since the last use and several of the measured sub-acute effects cognitive impairment day 1 (*r* = .29, *p* < .05), day 5 (*r* = .30, *p* < .05), negative mood day 1 (*r* = .36, *p* < .05) and paranoia day 2 (*r* = .30, *p* < .05). Days since the last use of cannabis were therefore used as a covariate in all the remaining analyses.

**Table 2:** Substance use by group (M (SE))

Substance	Control (*N* = 25)	Mephedrone (*N* = 21)	Group difference
Cannabis (times per month)	11.44 (2.21)	16.72 (2.30)	*t*(44) = 1.65, *p* = .11
Ecstasy (times per year)	23.32 (5.93)	24.05 (6.27)	*t*(44) = .08, *p* = .93
MDMA (times per year)	27.56 (6.50)	21.10 (6.27)	*t*(44) = .71, *p* = .48
Cocaine (times per year)	21.76 (4.06)	24.05 (3.88)	*t*(44) = .49, *p* = .69
Amphetamine (per year)	45.90 (35.78)	12.63 (6.32)	*t*(44) = .63, *p* = .53
Mushrooms (times per year)	1.50 (.38)	1.46 (.29)	*t*(44) = .09, p = .93
LSD (times per year)	1.50 (.29)	4.14 (3.12)	*t*(44) = .59, *p* = .57
Ketamine (times per year)	16.94 (2.78)	13.11 (5.30)	*t*(44) = .61, *p* = .55

**Table 3:** Participants’ mephedrone consumption history (M, range)

	Control (*N* = 25)	Mephedrone (*N* = 21)
Age at first use	20.00 (20.00–23.50)	23.00 (20.00–25.00)
Duration of use in months	12.00 (12.00–18.00)	12.00 (12.00–16.00)
Days since last use	7.00 (7.00–14.00)	7.00 (7.00–14.00)
Frequency of use per year	36.00 (24.00–48.00)	36.00 (15.00–52.00)
Usual dose per session (g)	1.00 (1.00–1.50)	1.50 (1.00–2.00)
Maximum dose per session (g)	2.00 (1.50–2.00)	2.00 (1.50–3.00)

Participants who did not opt to use mephedrone during the study were more likely to experience worse comedowns (67% of control vs 44% of mephedrone users agreed to the statement that “mephedrone has a bad comedown”, [*t*(44) = 3.53, *p* < .001]) but more likely to rate mephedrone as an enjoyable drug (74% of control vs 65% of mephedrone users agreed to the statement that “mephedrone is an enjoyable drug”, [*t*(44) = 2.19, *p* < .043].). More of the mephedrone group reported mephedrone dependence as according to DSM-IV (76 vs 60% of the control group, *χ*^2^(1) = 1.36, *p* = .24) based on endorsement of a minimum of three of the seven symptoms; however, the groups did not differ significantly. In regard to route of administration, all participants reported to snort mephedrone and never to have injected mephedrone.

### Mephedrone craving

Factor analysis of the Mephedrone Craving Questionnaire (MCQ) data yielded factor 1: eight items relating to expectancies of positive and (mainly) negative reinforcement; factor 2: eight items relating to an overwhelming and urgent desire to consume; factor 3: three items relating to mild desires and intentions to consume; and factor 4: three items relating to control over consumption of Mephedrone. At baseline, control participants reported elevated factor 1 scores [*t*(48) = 3.71, *p* = .001], factor 3 scores [*t*(48) = 2.86, *p* = .006] and factor 4 scores [*t*(48) = 2.60, *p* = .013] compared to the mephedrone group .

### Substance use during study

Mephedrone use during the study in the mephedrone group ranged from 0.5 to 2 g of mephedrone on day 2 (M = 1.03, SD = .37). There were no group differences in the extent of subsequent use of cigarettes, ecstasy, cocaine, LSD, ketamine, poppers or other substances. The majority of participants smoked cigarettes throughout the study; no significant differences were observed between the two groups. Use of other substances throughout the study was not frequent enough to analyse the results. Two individuals in the mephedrone group and one in the control group also consumed ecstasy, one in the mephedrone group and one in the control group consumed cocaine, and two participants in the mephedrone group also consumed ketamine. As to test whether there were significant differences between these and other participants, they were removed from the analyses and then added again, and the results were compared. No significant differences were observed.

On day 1, all participants in the mephedrone group consumed alcohol and all but seven in the control group (mephedrone M = 15.43 units, SD = 4.28; control M = 8.96, SD = 7.95; *t*(44) = 3.34, *p* < .01). Alcohol was thereafter consumed by participants in both groups, throughout the study; no significant differences were observed. Participants in the mephedrone group consumed significantly more cannabis throughout the study than did those in the control group. Throughout the study, nine participants in the control group and three in the mephedrone group never consumed cannabis. Significant differences of cannabis use among mephedrone users and controls (respectively) were observed on day 1 (M = 1.81, SD = 1.96; M = .32, SD = .80), day 4 (M = .86, SD = 1.39; M = .20, SD = .71), day 5 (M = 1.19, SD = 1.33; M = .28, SD = .54) and day 6 (M = 1.76, SD = 1.57; M = .40, SD = 1.19); all at *p* < .01.

Participants in the mephedrone group also reported more cannabis use than control participants across days [*F*(1, 44) = 11.26, *p* < .01] and more restless sleep [*F*(1, 44) = 9.82, *p* < .01]. Alcohol use on day 2 was also correlated with mean negative mood [*r* = .38, *p* < .05; *r* = .45, *p* < .01]. Therefore, cannabis and restless sleep were treated as covariates in all the remaining analyses, and alcohol use on day 2 was treated as a covariate with mood. As alcohol consumption also differed between the groups on day 1, it was used as a covariate in all the remaining analyses.

### Sub-acute effects of mephedrone

Responses on the four DRS items (“how depressed did you feel?”; “how irritable and crabby did you feel?”; “how ruminating and brooding were you about negative things?” and “how anxious did you feel?”) were highly positively correlated [*p* < .001] and were pooled to provide a single negative mood score. The two DRS items (“how difficult was it for you to concentrate on things?” and “how difficult was it for you to remember things?”) were highly correlated [*p* < .001] and were also pooled to provide a single subjective cognitive impairment score. The two DRS items (“how much do you want to take Mephedrone right now?” and “how much have you been thinking about taking Mephedrone today?”) were highly positively correlated [*p* < .001] and were pooled to provide a single post-baseline mephedrone craving score.

**Fig. 1:**
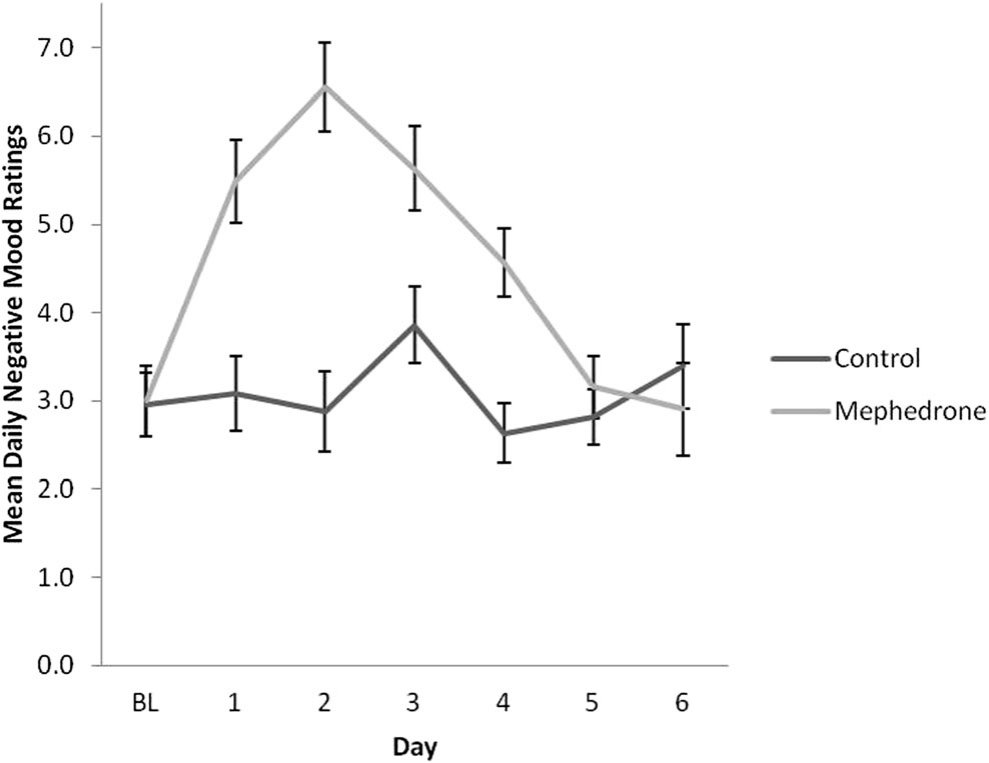
Ratings over time of negative mood for sub-acute effects of mephedrone and control group

**Fig. 2:**
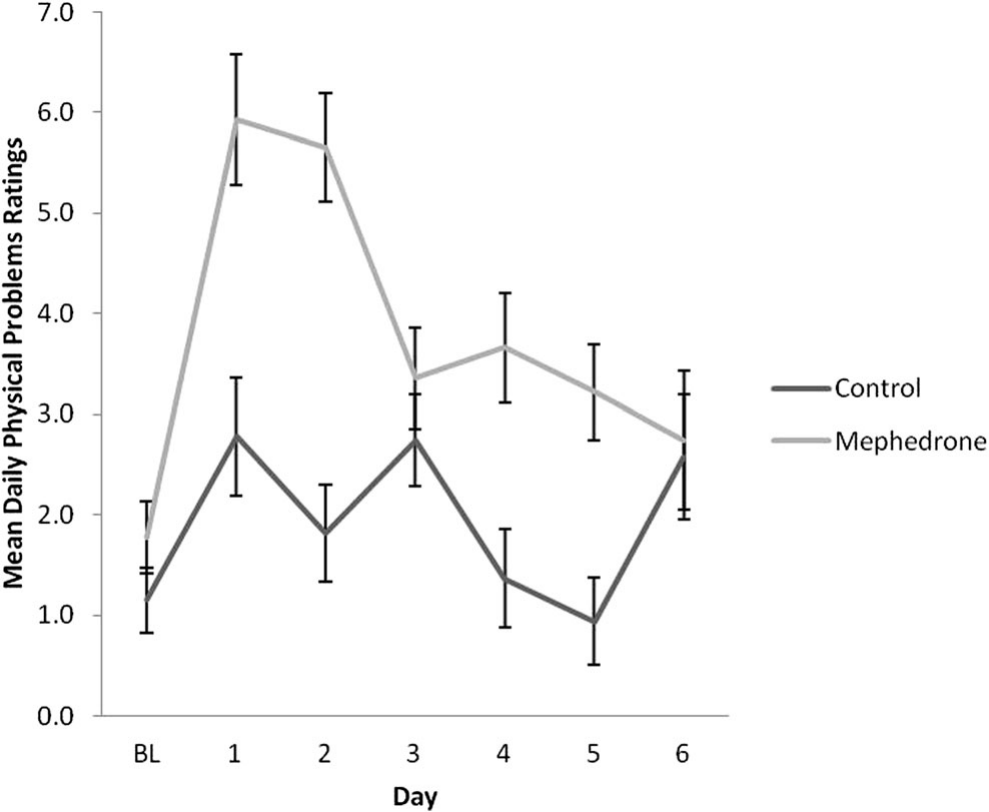
Ratings over time of physical problems for sub-acute effects of mephedrone and control group

**Fig. 3:**
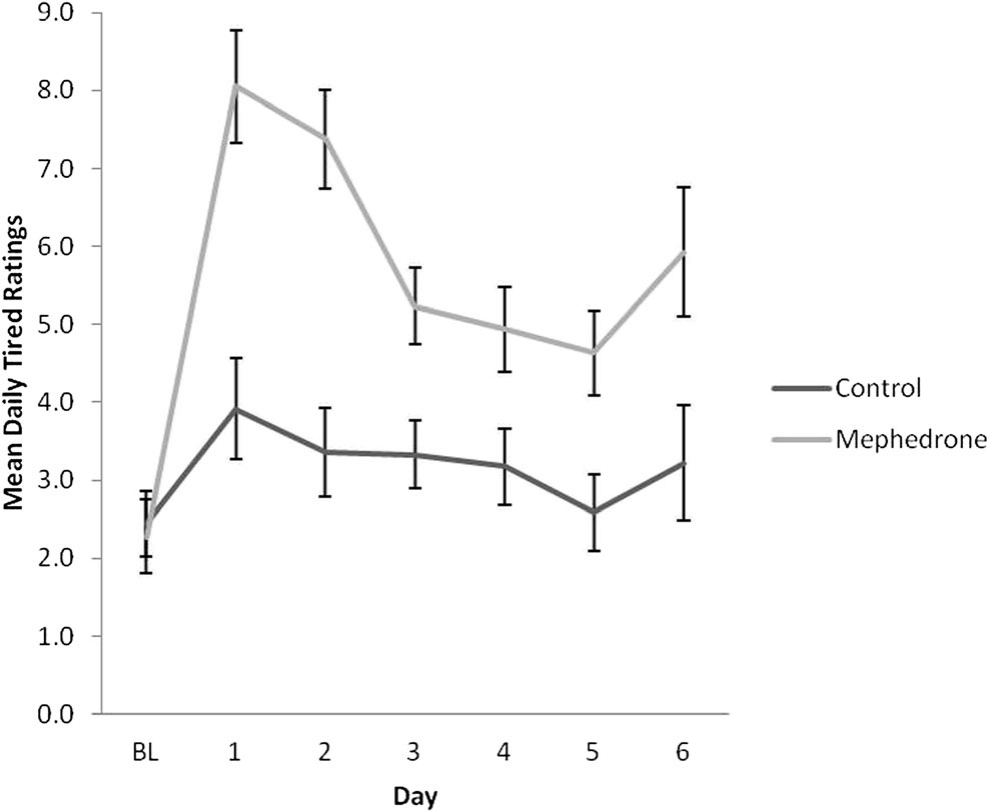
Ratings over time of tiredness for sub-acute effects of mephedrone and control group

**Fig. 4:**
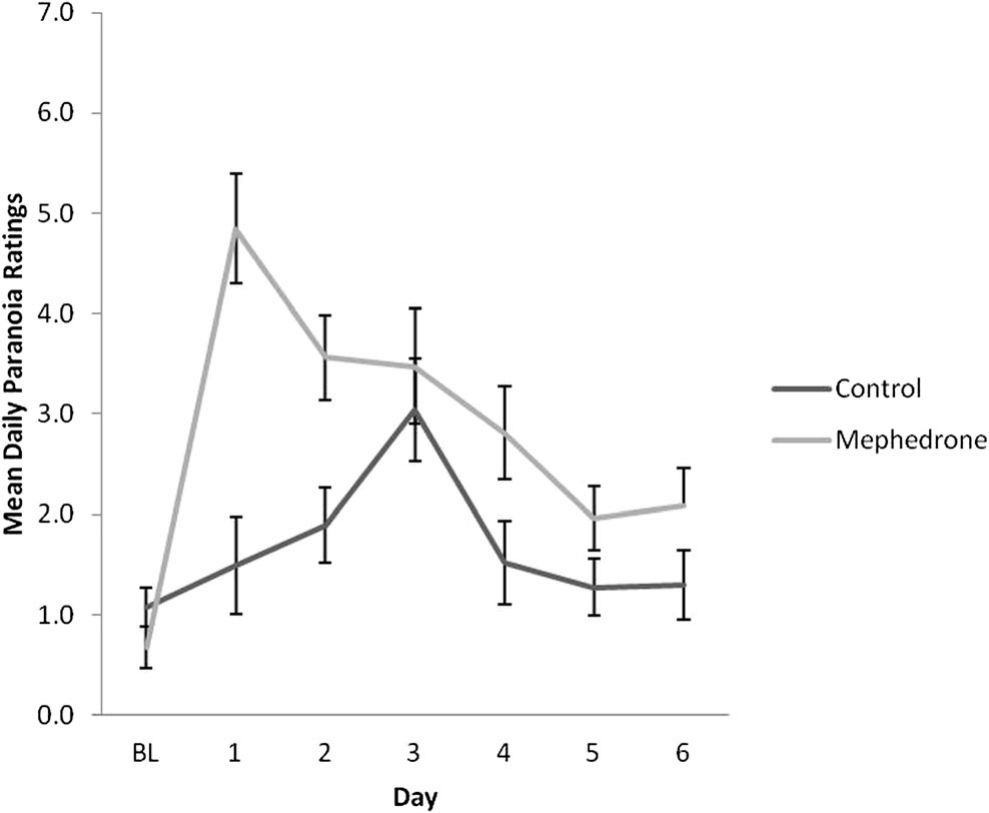
Ratings over time of paranoia for sub-acute effects of mephedrone and control group

**Fig. 5:**
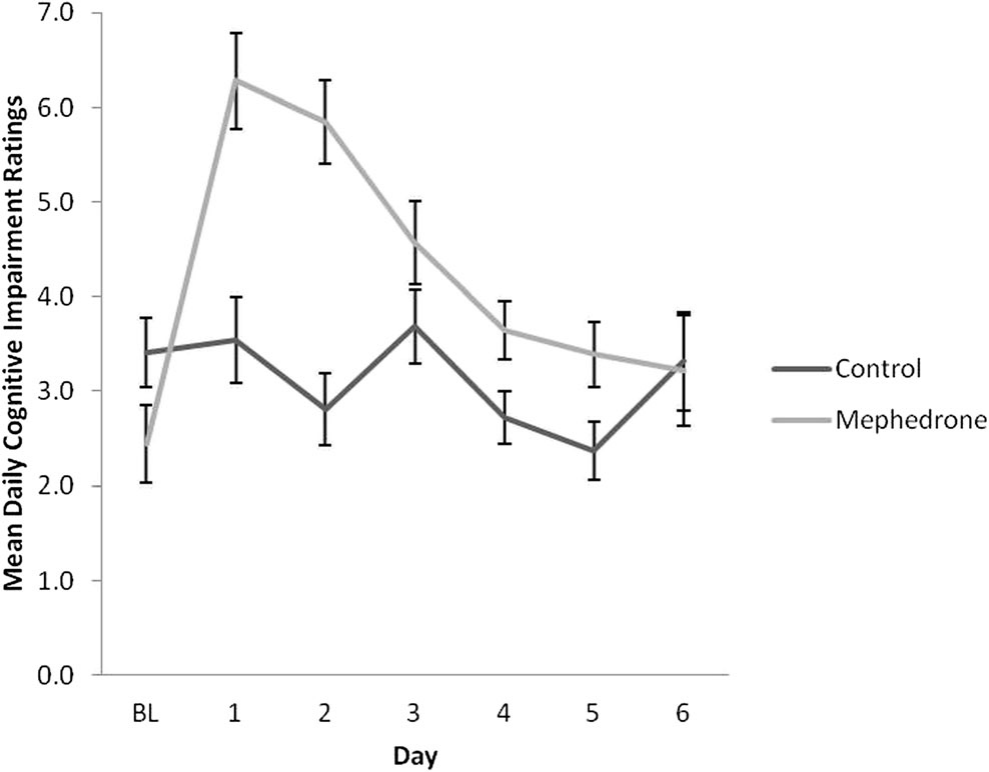
Ratings over time of cognitive impairment for sub-acute effects of mephedrone and control group

Baseline measures of each scale were not significantly different across groups. Results of repeated measures ANOVA for each DRS scale are presented in Table 4. Participants who opted to take mephedrone reported significantly elevated negative mood for a minimum of 4 days after use (Fig. 1), increased physical problems across days (Fig. 2) and increased tiredness across days, in particular, on day 2 after use (Fig. 3). All symptoms remained significant before and after controlling for covariates. Participants who used mephedrone also reported increased levels of paranoia, in particular, on day 2 after use (Fig. 4), and impaired cognition for at least 2 days after use (Fig. 5). However, these results were not significant after controlling for covariates. The mephedrone craving-related items did not differ significantly between groups across days. No significant differences across groups were observed for self-reported hunger.

**Table 4:** Significant group differences of DRS over time, results of repeated measures ANOVA, with and without covariates

Measure	Excluding covariates	Including covariates
Negative mood	*F*(1,43) = 6.10, *p = .03*	*F*(1,41) = 5.08, *p = .04*
Cognitive impairment	*F*(1,41) = 5.15, *p = .04*	*F*(1,41) = 3.18, *p* = .08
Mephedrone craving	*F*(1,41) = 1.27, *p* = .19	*F*(1,41) = 0.96, *p* = .37
Hunger	*F*(1,41) = 1.34, *p* = .48	*F*(1,41) = 1.15, *p* = .59
Physical problems	*F*(1,44) = 7.15, *p = .002*	*F*(1,43) = 9.98, *p = .002*
Tiredness	*F*(1,43) = 8.49, *p = .002*	*F*(1,42) = 11.52, *p = .003*
Paranoia	*F*(1,42) = 9.29, *p = .003*	*F*(1,42) = 9.29, *p* = .07

The items which are of significance are italicized

## Discussion

The present study is the first empirical investigation into the sub-acute effects of mephedrone in regular mephedrone users, where effects were measured daily throughout a week and where several baseline and concurrent factors were controlled for. About half the sample voluntarily consumed mephedrone during the testing phase. At baseline, the two groups did not differ on psychopathology, personality or past and current substance use. Additionally, the two groups were closely matched on prior mephedrone use. When controlling for co-use of cannabis, alcohol and restless sleep, mephedrone use produced significant sub-acute effects. Negative mood was markedly elevated for 4 days after mephedrone use while physical problems (aches/pains etc.) and fatigue were all significantly elevated for at least 2 days after use.

Psychopathology, impulsivity, delayed discounting, substance use, alcohol problems and demographics did not differ significantly between those who opted to consume mephedrone and those who did not. This indicates genuine mephedrone effects rather than effects that stem from psychopathology or past substance use. This is not in line with previous studies where participants who chose to consume a substance during the testing period also reported higher levels of psychopathology (Parrott et al. 2000; Huxster et al. 2006). For example, Huxster and colleagues found elevated global psychopathology in ecstasy users. The present findings may suggest that our sample was more homogeneous than in previous studies.

Nevertheless, some differences were observed. Participants who opted not to take mephedrone during the study also reported higher levels of cravings for mephedrone at baseline as well as worse comedowns and a higher level of enjoyment when consuming mephedrone. It is possible that participants who opted not to take mephedrone already knew they would not. These findings are in line with Tiffany’s model of craving (Tiffany 1999). According to this model, thoughts, actions or associations to substance use (for example questions about the substance as in the questionnaires administered) activate substance use action schemata. If these schemata are blocked (for example, due to not being able to use, not having access to the substance or choosing not to use (possibly due to past negative experiences)), cravings occur. Furthermore, our results suggest that use during the study was not associated with psychopathology, impulsivity, past use, dependence or other demographics. Other potential differences which determine whether participants chose to take a substance or not were not included and therefore outside of the scope of this discussion. It would however be of interest for future studies shed light on this as it may impact subjective experiences.

Significant sub-acute effects of mephedrone were observed on negative mood, fatigue and physical problems. Symptoms increased markedly within the first 24 h after mephedrone consumption and decreased thereafter. The results are similar to other studies on sub-acute effects of MDMA in regard to cognitive impairment and negative mood (Curran and Travill 1997; Parrott and Lasky 1998; Verheyden et al. 2002; Curran et al. 2004; Huxster et al. 2006; Freeman et al. 2011). Parrott and Lasky (1998) study further supported these findings as well as strengthened them by chemically verifying the presence of MDMA in the participants’ saliva (Parrott et al. 2008).

However, some of these studies did not control for confounding factors such as sleep deprivation (Verheyden et al. 2002; Curran et al. 2004). The present study shows that these effects are still valid after controlling for sleep deprivation and other co-use of substances.

The present findings on the sub-acute effects of increased physical problems support cross-sectional and retrospective results (Prosser and Nelson 2012; Karila et al. 2016) and are in line with findings on sub-acute effects of amphetamine and MDMA in the form of loss of appetite, sleeping difficulties and bruxism (Verheyden et al. 2003). Sub-acute effects of paranoia differed significantly across the groups but were not significant after controlling for cannabis, alcohol use and sleep, suggesting that paranoia is likely to be a symptom of these factors (Kahn-greene et al. 2007), or alternatively, a symptom of the combination of the confounders as well as mephedrone. Additionally, sub-acute effects of mephedrone indicated significantly heightened levels of cognitive impairment. However, when covariates were controlled for, this finding was no longer significant. This is also in line with other studies on the sub-acute effects of MDMA on cognitive impairment which after also controlling for sleep and other substance use no longer found significant effects (Huxster et al. 2006). It is possible that this is explained by that sleep deprivation, or possibly sleep deprivation combined with the substance, causes an increase in cognitive impairment, rather than the actual substance in itself (Lim and Dinges 2010). The present study shows the importance of controlling for other possibly confounding factors as some symptoms, such as increased levels of paranoia and cognitive impairment, may not only be due to the substance in question. This is important when performing naturalistic studies where polydrug use is the norm rather than the exception.

Although the design of this study allowed us to control for differences between groups at baseline and other subsequent substance use, the main limitation was our use of exclusively subjective measures. While mood, impulsivity, craving and personality are all typically recorded with self-report measures, it would have been beneficial to confirm substance consumption via a biological measure. However, the study was performed in 2010, just before the status of mephedrone moved from legal to illegal. As it was not possible in the present study to confirm use via biological samples, we found reassurance in that assays of mephedrone in circulation at the time of the present study indicated high purity (Gibbons and Zloh 2010) and self-reported recreational substance use has been reported to show good concordance with laboratory verified use (Sobell et al. 1995; Thomasius et al. 2003; Solbergsdottir et al. 2004; Pirona and Morgan 2010). Nevertheless, it would have been ideal to perform urinalyses of participants to confirm the accuracy of self-reported substance use during the testing phase. Additionally, it is possible that symptoms measured may be influenced by other life events. For example, paranoia peaked on day 3 among participants in the control group. Day 3 was a Monday which may be associated with the increase in paranoia due to going back to work/studies; it is also possible that other events on this day may cause the increase. We therefore recommend future studies to additionally record other daily events. Finally, the present study only asked whether participants sleep was restless. It may additionally be informative to also record hours of sleep in order to measure sleep deprivation as this may have more of an impact on the sub-acute effects presently measured.

It should be noted that a drug-naive control group would not be appropriate when employing this design as using a drug-naive control group would not control for possible effects of previous and/or chronic use. Additionally, an RCT design was not applied as it was not considered ethical to instruct participants to consume the substance. The present study was therefore of an observational design.

In conclusion, the present study provides further evidence of adverse sub-acute effects of mephedrone. After controlling for restless sleep at baseline and subsequent co-use of alcohol and cannabis, the sub-acute effects of mephedrone remained apparent, including persistent negative mood, physical problems and fatigue. Importantly, the present design allowed us to differentiate the genuine sub-acute effects of mephedrone from its chronic effects or sub-acute effects of other substances. These sub-acute effects appear to be similar to, but more marked than, those reported for ecstasy (Huxster et al. 2006). Thus, the present study provides the first clear indication of the extent and magnitude of the specific sub-acute effects of mephedrone in regular recreational users.
